# Improvement in Purity of Healthy Tomato Seeds Using an Image-Based One-Class Classification Method

**DOI:** 10.3390/s20092690

**Published:** 2020-05-08

**Authors:** Jannat Yasmin, Santosh Lohumi, Mohammed Raju Ahmed, Lalit Mohan Kandpal, Mohammad Akbar Faqeerzada, Moon Sung Kim, Byoung-Kwan Cho

**Affiliations:** 1 Department of Biosystems Machinery Engineering, College of Agricultural and Life Science, Chungnam National University, 99 Daehak-ro, Yuseong-gu, Daejeon 341–34, Korea; yasminjannat@o.cnu.ac.kr (J.Y.); santosh123@cnu.ac.kr (S.L.); mrahmed@o.cnu.ac.kr (M.R.A.); lalitm85@cnu.ac.kr (L.M.K.); akbar2016@o.cnu.ac.kr (M.A.F.); 2 Environmental Microbial and Food Safety Laboratory, Agricultural Research Service, U.S. Department of Agriculture, Powder Mill Rd. Bldg. 303, BARC-East, Beltsville, MD 20705, USA; Moon.kim@ars.usda.gov

**Keywords:** seed quality, machine vision, image processing, feature selection, GLCM features

## Abstract

The feasibility of a color machine vision technique with the one-class classification method was investigated for the quality assessment of tomato seeds. The health of seeds is an important quality factor that affects their germination rate, which may be affected by seed contamination. Hence, segregation of healthy seeds from diseased and infected seeds, along with foreign materials and broken seeds, is important to improve the final yield. In this study, a custom-built machine vision system containing a color camera with a white light emitting diode (LED) light source was adopted for image acquisition. The one-class classification method was used to identify healthy seeds after extracting the features of the samples. A significant difference was observed between the features of healthy and infected seeds, and foreign materials, implying a certain threshold. The results indicated that tomato seeds can be classified with an accuracy exceeding 97%. The infected tomato seeds indicated a lower germination rate (<10%) compared to healthy seeds, as confirmed by the organic growing media germination test. Thus, identification through image analysis and rapid measurement were observed as useful in discriminating between the quality of tomato seeds in real time.

## 1. Introduction

On a global scale, tomato is one of the most important vegetable crops, accounting for approximately 15% of the total vegetable production. Tomatoes are sensitive and prone to diseases, and over 20 bacterial and fungal diseases can affect tomato production. This requires the use of chemicals and pesticides to prevent the invasion of germs [1,2]. The good housekeeping team (GH, New York, USA) annually compiles a list of fruit and vegetables with the highest levels of pesticide residues. In the list compiled for 2019, tomatoes were at number 10 [3], which is an alarming issue for the climate and mass health. The major constraints in tomato production include viruses, pests, diseases, and a lack of quality seeds for further healthy tomato production [4,5,6,7] which affect the consumer, seed-producing industries, and the economy. Seed pathogens can cause germination failure and transmit diseases from the seed to the seedling and fruit [8,9]. Thus, non-contaminated seeds should be planted to increase crop yield and secure future seed production of tomatoes. Moreover, tomatoes produce a small number of seeds (150–300 g/ha based on the variety) [10], thus rendering healthy tomato seeds expensive.

Producing disease-free and reputable plants and healthy seeds is necessary owing to their high value. High-quality seedlings require high-quality seeds, which can be defined by four components: genetic, sanitary, physical, and physiological factors. Seed quality evaluation is essential at the beginning of planting to ensure the desired seedling population. Manual identification of high-quality seeds is slow and lack accuracy. The screening of healthy seeds is a basic tool for ensuring secure food supply and is necessary for two principal reasons: quality and quarantine. The commercial value of the seeds decreases if they are infected, even though seeds are free from quarantined objects [11]. Seeds are obtained from the field, and thus there is a high possibility of mixing foreign materials, such as broken seeds and non-seed items (i.e., stem, straw, and leaf material), and other crop seeds with desired seeds. The contaminants must be removed before planting the seeds and are difficult to determine visually. A recent report on seed screening indicated that more than 250 screens in perforated steel or wire cloth with air screening are adopted to screen seeds [12]. More than two screens are used to separate the seeds from foreign materials, and this is a time-consuming task. A small unit of seeds can be tested efficiently in laboratories. However, this method requires special arrangements and many personnel, which is potentially unavailable in certain countries [13]. Thus, it is essential to ensure rapid certification of seeds with high efficiency and accuracy. For technical and economical purposes, it is important to implement a machine vision-based method that is fast, reliable, and accurate.

Given non-destructive methods of seed quality evaluation, previously published studies indicated the increasing use of image processing and spectral techniques in the agriculture sector. Additionally, there is an increase in the non-destructive experimentation of agro-food materials including machine vision [14], X-ray techniques [15,16,17], hyperspectral imaging [18,19,20], and Fourier transform near-infrared (FT-NIR) spectroscopy [21,22,23] due to improvements in computation power. The machine vision system is adopted in numerous food and agribusiness systems to provide rapid, robust, and reliable results. A more detailed description of imaging technologies for non-destructive measurement was given by Ahmed et al. [24]. Peterson et al. indicated that seed classification accuracy can be improved by using color images [25]. Image-based studies to discriminate between different varieties of rice grains [26,27], automatic classification of barley, oat, and rye [28], phenotyping of individual seeds [29], germination prediction of rice [30], and quality assessment of tomato seed [31,32] provide alternatives to manual inspection. However, the studies only focused on identification of species and not on the identification of healthy seeds or characterization of infected seeds along with inert materials. Recent studies on machine vision techniques for physical purity in relation to germination for soybean seed [33] and oats characterization [34] revealed the increasing use of the machine vision approach in agriculture.

The proposed study attempted to address a machine vision technique and image processing method to discriminate between infected (black-dotted) tomato seeds and inert materials (broken seeds and non-seed items) from healthy seeds. The study is organized as follows. First, the hardware to capture seed images was described. Furthermore, features (color, morphological and textural) of the seeds were selected for model calibration. Finally, the multivariate (one-class classifier, OCC) approach was applied in the classification to distinguish healthy seeds from the infected seeds with inert materials. The proposed study comes up with an identification system for healthy tomato seeds based on their properties. From a practical viewpoint, the proposed technique can offer an efficient and cost-effective alternative that can provide rapid and real-time seed sorting, and this will lead to a high yield.

## 2. Materials and Methods

### 2.1. Samples

**Figure 1:**
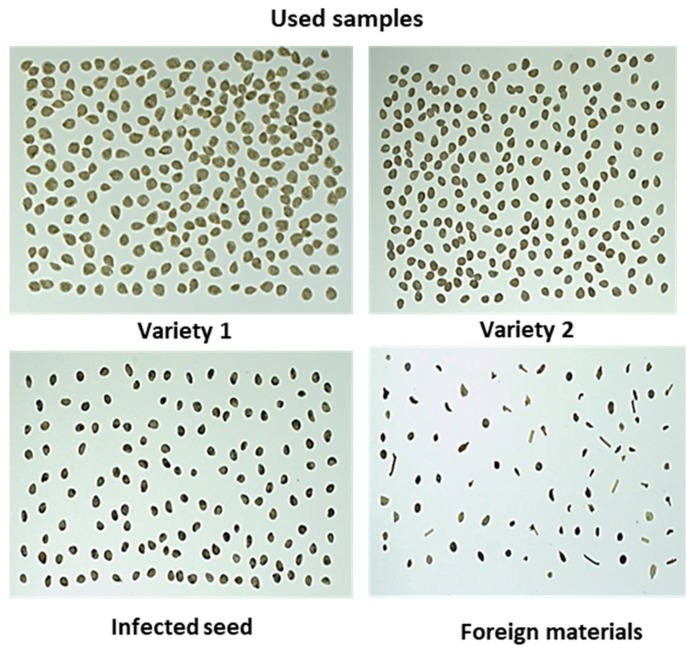
Samples used to build the model.

The samples were provided by a seed company (Nongwoo-bio INC, Gyeongi-do, Korea) that included; two different varieties commercially termed as Variety 1 and Variety 2 (in the study it will be mentioned further as V1 and V2) of healthy tomato seeds, black-spotted tomato seed varieties, and different inert materials. It was assumed that tomato seeds with black spots were infected (non-viable), and this was subsequently confirmed by the organic growing media germination test. The provided tomato seeds were mixed with different types of naturally occurring foreign materials with different attributes (i.e., size, shape, and color, such as stem and other crop seeds similar to tomato seed and broken stones) and abnormal seeds (i.e., darker color and irregular shape, and broken seeds, illustrated in Figure 1). The infected seeds were selected manually by the seed experts from the seed providing company. However, the inert materials were manually selected in the laboratory from the seed considering the visually different criteria of inert materials, and were confirmed by the seed experts before their use in this work.

All the seed samples were stored in a small room that was especially designed for seed batches by following the rules of the Korean seed and variety service (KSVS) authority in the seed company and at a controlled temperature of 5 °C in a sealed plastic container.

### 2.2. Imaging System

**Figure 2:**
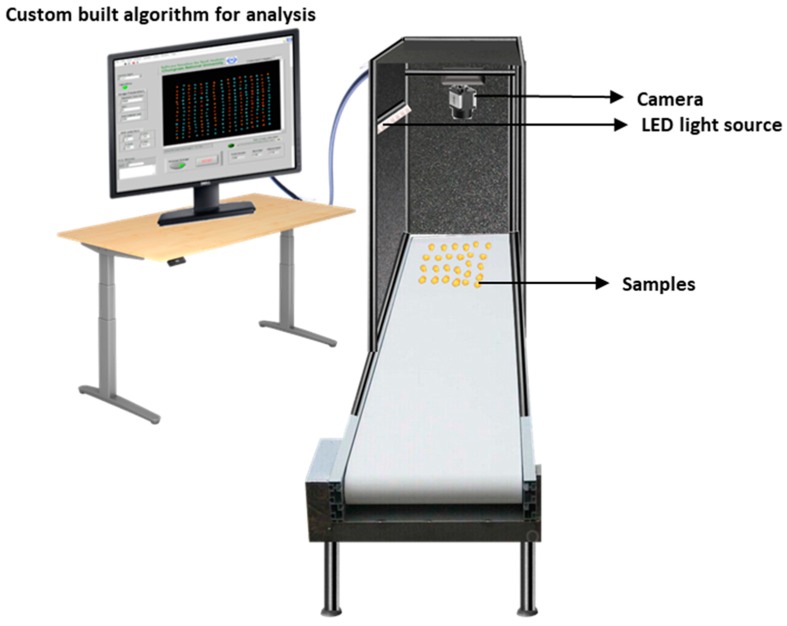
Schematic diagram of image acquisition for samples.

Figure 2 shows the image acquisition system that is used to capture the images of the samples. Seed samples were captured with a complementary metal oxide semiconductor (CMOS) USB 3.0 camera (MV-CA050-20UC, HIKVision, MA, USA) with a CMOS detector for each color channel. The optical axis of the 25-mm lens system was set as perpendicular to the black background of a custom-built frame. Illumination was provided via a white light emitting diode (LED) light source that was mounted on a side at an angle of 30° with respect to the optical axis. The LED stripe was containing 15 lights in a single row with a power source of 12 V, and the power consumption for each light was 6 W. The camera and illuminating light were connected at a distance of 55 cm from the platform where the seeds were placed. To decrease the outside light effect, the entire frame was covered from each side with black surfaces during image acquisition. The camera was calibrated for color matching by using a simulated “ColorChecker” chart to validate image colors to estimate color variations against known target values. The patches of the color chart were detected irrespective of their position and orientation. The white and dark patches of the color chart were used to estimate the color variations and to compute the red (R), green (G), and blue (B) component values, and the maximum allowed value was 255. All images were captured via the field of view (FOV) and covered approximately 16 cm × 22 cm. This arrangement ensured that the camera was color calibrated and corresponded to the preferred FOV. A white platform was used to clearly identify slight color or textural changes of the seeds. Images with small textural details were obtained perfectly using the settings.

During capturing the images some samples were barely touched or overlapped on the sample platform. The proposed algorithm for this computer vision system can separate each seed regardless of touching or overlapping from other materials (broken seed or inert materials) without any human intervention. Subsequently, overlapped seeds were separated into a single seed by using a customized algorithm. This aspect is beneficial in case of working with huge seed bunches because it is not always possible to manage the positioning of seeds without touching or overlapping.

### 2.3. Image Acquisition

In this study, images of healthy tomato seeds, infected seeds, and foreign materials were captured in various ways. First, images of healthy tomato seed varieties, foreign materials, and infected seeds were captured only containing one single group. After that, samples were set in orientated lines as healthy seed varieties, foreign material, and infected seeds and then captured the images. In addition, finally, all the samples were mixed manually in different orientations and captured the images. These final images were used to build the model with model validation.

A total of 3000 good tomato seeds from two varieties (1500 seeds of each variety) were captured in the images. Among them, 2000 good seeds (1000 seeds of each variety) were used to develop the model, and the remaining seeds were used as the test set to validate model accuracy. The obtained dataset for samples was composed of 30 images where 21 images were used to build the calibration model, and the model performance was validated from the remaining nine images.

### 2.4. Image Analysis and Pre-Processing

**Figure 3:**
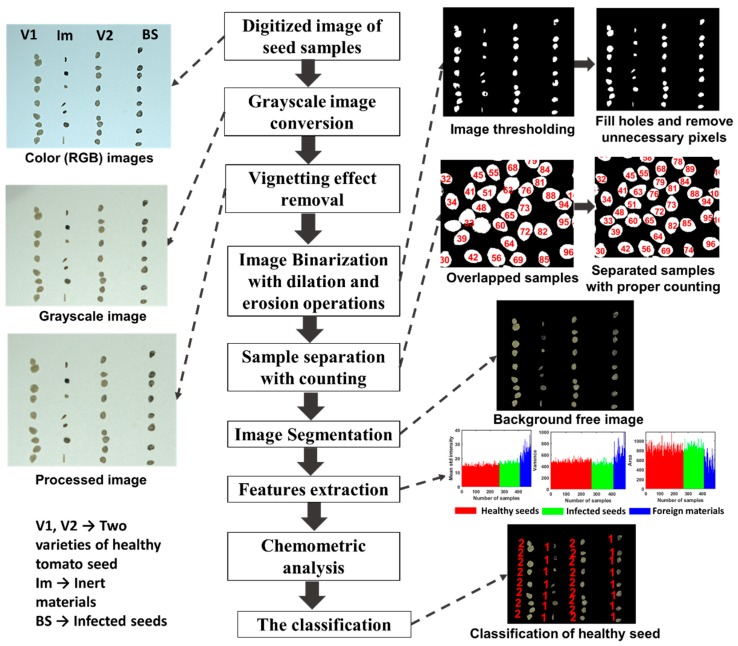
The phases of the proposed approach.

In this section, a custom-built algorithm was used to analyze the images with several steps were followed and the workflow is shown in Figure 3. From the color (RGB) images, first the radial fall-off intensity from the center were removed and produced binary images of uniform exposure (discussed in detailed in Section 2.4.1). To produce binary image, a threshold value was calculated by converting RGB color image to gray scale image. We found that samples are always dominated in the area of gray levels less than 170 and for the background the gray level was higher than 170. Therefore, in this work, the threshold value was set as 170 and worked well as the same camera settings, background, and illumination. From the resultant binary images, unwanted pixels were removed, the overlapped samples were separated programmatically, and thus the denoised images were produced. The denoised binary image was then multiplied to original color (RGB) images. Hence, features would be collected from the images including color feature, it was carefully noticed that the color information would retain same during the segmentation process. All analyses of the seed classification model including image processing (feature selection and classification approach) for the tomato seeds were performed with a custom-built algorithm based on MATLAB (2018a, MathWorks, Natrick, MA, USA).

#### 2.4.1. Vignetting-Effect Removal

Generally, a captured image can exhibit a radial fall-off intensity from the center, and this is known as the “vignetting effect”. Captured images with a uniform exposure and without a vignetting effect are the preliminary concerns of image processing. The vignetting effect is common in optics (LED- or halogen-based illumination) and can affect the result. Therefore, the vignetting effect should be removed from images before starting the analysis. The vignetting effect can be produced due to multiple factors although it is mainly proportional to the aperture and disproportionate to the focal length of the lens [35].

**Figure 4:**
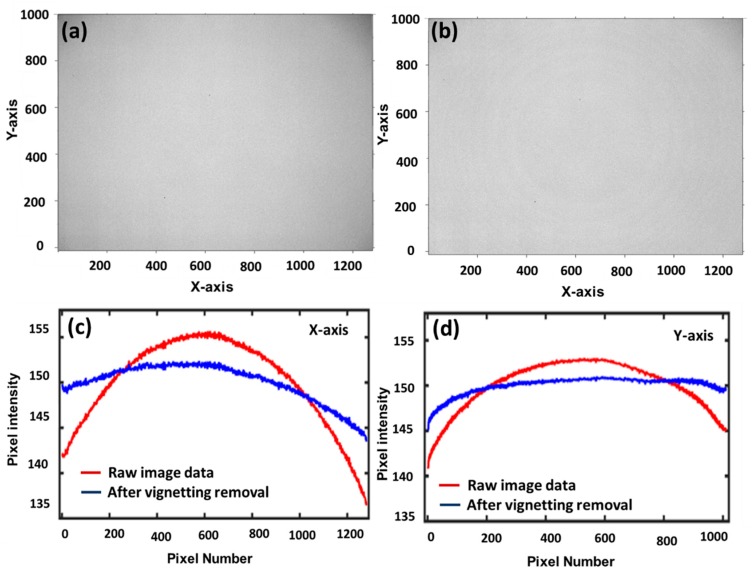
Vignetting effect and removal for images. (**a**) Raw image of white reference shows high intensity at the center and dark corners are seen clearly; (**b**) Corrected image shows almost uniform intensity; (**c**) Intensity corrected for X-axis; (**d**) Intensity corrected for Y-axis.

To prevent the vignetting effect, the position of the FOV was optimized by fixing the focal length and focus distance, which were used to capture images. Furthermore, an algorithm developed by Zheng et al. [36] was applied to mitigate the vignetting effect of captured images in MATLAB. As shown in Figure 4a,b a significant exposure variation due to the vignetting artifact was removed via image processing. The removal of the vignetting effect was further emphasized in the axes after removing the vignetting effect (shown in Figure 4c as the blue solid line). It was observed that the central area of the image was nearly flat after removing the vignetting effect when compared to raw images (in Figure 4d red line).

#### 2.4.2. Feature Extraction

For the calibration image dataset, features from two varieties of healthy tomato seeds were extracted. Three sets of features, namely color features, morphological features, and textural features, were used to evaluate the features of normal seeds relative to bad or infected tomato seeds with foreign materials (broken seed and non-seed items). The color feature was used to differentiate the infected (black-spotted) seeds from the good seed. Furthermore, some foreign materials were also of a different color from the good seed variety. Several morphology and texture-based analyses were performed to differentiate good seeds from foreign materials.

All the features were extracted after removing the background of the RGB image. The errors in image thresholding were corrected via morphological opening, closing, erosion (removed pixels on object boundaries), and dilation (added pixel of object boundaries) operations after binarization. Finally, the seed pixels were mapped after watershed segmentation to separate touched and overlapped seeds in the image. When the seeds were segmented properly, they were labeled using the developed algorithm, based on seed centroids and the background free image was extracted for further analysis. Finally, a labeled matrix was formed to obtain pixel coordinate data for each sample, which was aggregated in a separate data structure.

The first set feature is a color histogram, which is one of the most well-known color features used for image feature extraction. If the color distribution of samples is characterized by its moments as a probability distribution, then color distribution information is also characterized. In the study, color moments (mean and variance) were used to compare two samples and is a relatively new approach in color indexing [37]. In previous studies, the mean and variance were proven as efficient and effective in color distribution of images [38].

To analyze the image spatial structures, morphology analysis is a powerful technique as it aims in analyzing the shape and form of object [39]. After sample segmentation in the image, several measurements were performed and investigated. The gray levels co-occurrence matrix (GLCM) was selected as the third set feature extraction algorithm and is an effective statistical technique for image classification and texture analysis [40,41] to differentiate samples texture. Textural features are used to segment images into the desired image regional area (ROI) and classify the regions. The GLCM approach is a tabulation of the way frequent combinations of pixels occur in an image or an image section. In the study, the GLCM algorithm was used to predict healthy tomato seed texture with different inert materials. The advantage of the algorithm is that it considers the spatial distribution of the intensity of the gray level in the neighborhood from an image. Thus, the algorithm calculates a pair of pixels with specific values that occur in an ROI and then determines statistical parameters from the matrix. It also measures the intensity variation of the pixels from the ROI. Typically, the calculation is obtained with the following two parameters: (1) relative distance between a pixel pair and (2) their orientation. To extract the textural features, the GLCM algorithm was used. The algorithm called “GLCM_Features” was employed for the textural feature selection of the samples that create a GLCM. The function creates a GLCM by calculating how often a pixel with the intensity (gray level) value i occurs in a specific spatial configuration to a pixel with value j. Each element (i.j) in the resultant GLCM is simply the sum of the number of times that the pixel with value i occurred in the specified spatial relationship to a pixel with value j in the input image. After creating the matrix of GLCM, numerous statistical deviations can be calculated for the feature extraction. In this study, intensity statistics-based feature (energy) and texture-based features (correlation, homogeneity and contrast) provided important information for the identification of texture difference. In each measurement it contained four values due to the displacement vector of d which was constant for each sample and used four angles (θ) for d = 0, 45, 90 and 135 [42]. For further information about GLCM features please refer to [43,44,45].

**Figure 5:**
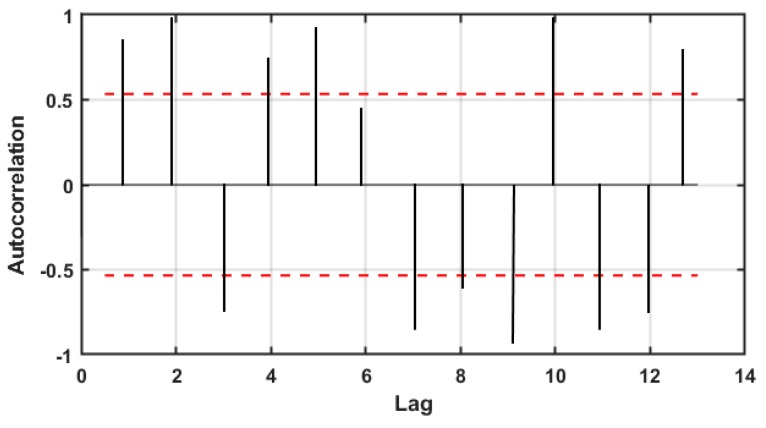
An autocorrelation plot showing the correlation between features.

To differentiate healthy tomato seeds from inert materials such as abnormal seeds (broken, darken and irregular shaped seeds) and foreign materials (other crop seeds and non-seed items), the samples showed significant inter-group variations including shape, size, and color variation. The selected features were divided into three categories; color, morphological, and textural feature and accumulated in a matrix. After that, the correlation between the extracted features was calculated. Generally, an autocorrelation plot is designed to observe whether the elements of a series are positively correlated, negatively correlated, or independent to each other. Here, the value of the autocorrelation function on the vertical axis was in a range from −0.5 to 0.5. Each spike that rises above or falls below the dashed lines is considered to be statistically significant. This means the spike has a value that is significantly different from zero. If a spike is significantly different from zero, that is evidence of autocorrelation. A spike that is close to zero is evidence against autocorrelation. Features which were close to zero were not granted for further analysis. Therefore, the best 14 features illustrated in Figure 5 (2 color, 8 morphological and 4 textural features) were identified and applied to detect healthy tomato seeds from bad seeds and foreign materials.

### 2.5. Analysis of Variance Test for Tomato Seed Varieties

This statistical analysis was performed between the two varieties of healthy tomato seeds to determine feature differences. In the study, only one independent variable was considered. The null hypothesis was that the means of all levels are equal, and the alternative hypothesis was that the means of one or more levels are different. If the value of p becomes less than 0.05 (p ≤ 0.05), the null hypothesis would be rejected. Otherwise for the alternative hypothesis it would be rejected. The analysis was performed using MATLAB (2018a, MathWorks, Natick, MA, USA).

### 2.6. One-Class Classification for the Identification of Healthy Tomato Seed

Recently, the OCC approach gained increasing attention in the machine learning field [46,47], and the main focus is to recognize instances of a concept by only using examples of the same concept [48]. In the classification method, only a single object class (i.e., good seeds from both varieties) were used as the training set, and all the other classes that excluded the training set were considered alien or outliers. OCC is different from the binary classification in which the data of the second class is absent in the training set. The OCC is effectively used in multiple real-world problems in the machine vision approach [49,50]. Data-driven soft independent modeling of class analogy (DD-SIMCA) is one of the most common and effective OCC techniques in chemometrics [51]. The main steps of the DD-SIMCA algorithm are briefly presented. First, the principal component analysis (PCA) decomposes the training (*I* × *J*) data matrix *X* as follows:

(1) $$X=TP^{t\;}+E$$

where *T* denotes the score (I×A) matrix, P is the loading (J×A) matrix, E denotes the matrix of residual (I×J), and A denotes the number of principal components (PCs). The total distance (score and orthogonal distances) for each training sample is calculated using the results of the PCA decomposition as follows:

(2) $$c=N_{h}\frac{h}{h_{0}}+N_{v}\frac{v}{v_{0}}\propto \chi^{2}\left(N_{h}+N_{v}\right)$$

where *h* and *v* denote various tolerance areas of scores (SD) and orthogonal distances (OD), respectively, *v*_0_ and *h*_0_ denote scaling factors, and $N_{h}\;$ and $N_{v\;}$ denote the number of degrees of freedom (DoF). The threshold or acceptance area for the training group is determined as follows:

(3) $$c\leq c_{crit}\left(\alpha \right)$$

where $c_{crit}=x^{-2}\left(1-\alpha ,N_{h}+N_{v}\right)$, *α* is the given type I error, and (1−α) denotes the quantile of the chi-square distribution with $\;(N_{h}+N_{v})$ DoF.

When the acceptance area is determined, the model is ready to classify new samples. The acceptance area in the orthogonal direction relative to the score distance is represented by *α*. The sample from the training set is qualified as “regular” if it is located inside the acceptance plot. The cut-off value, *γ*, is calculated to determine the “outlier” border of the model and depends on the size of the training set (I). The outlier area is formed as follows:

(4) $$\gamma =\left\{\left(h,v\right):N_{h}\frac{h}{h_{0}}+N_{v}\frac{v}{v_{0}}>\chi^{-2}\left({\left(1-\gamma \right)}^{1/l},N_{h}+N_{v}\right)\right\}$$

The “extreme” area is located between the “regular” and “outlier” areas. The *γ* value specifies the erroneous position of a regular object from the training set (I), and the greater the value of *I*, the further the position of the outlier area. For a more detailed description and algorithm modification, readers can refer to [52,53].

### 2.7. Evaluation of Germination of Tomato Seeds

After building the tomato seed identification model, an organic growing media germination test was performed for 420 tomato seeds (210 healthy seeds and 210 black-dotted seeds) by following International Seed Testing Association rules [54]. During the germination procedure, for the first two days, the seeds were stored in dark at 30 °C. After two days, the seeds were stored in 8 h of light at 30 °C followed by 16 h of darkness at 20 °C to reproduce field conditions. A sufficient amount of water was provided to ensure that the soil was moistened whenever necessary. The procedure was performed for 14 days and data was recorded.

## 3. Results and Discussion

### 3.1. Image Analysis

**Figure 6:**
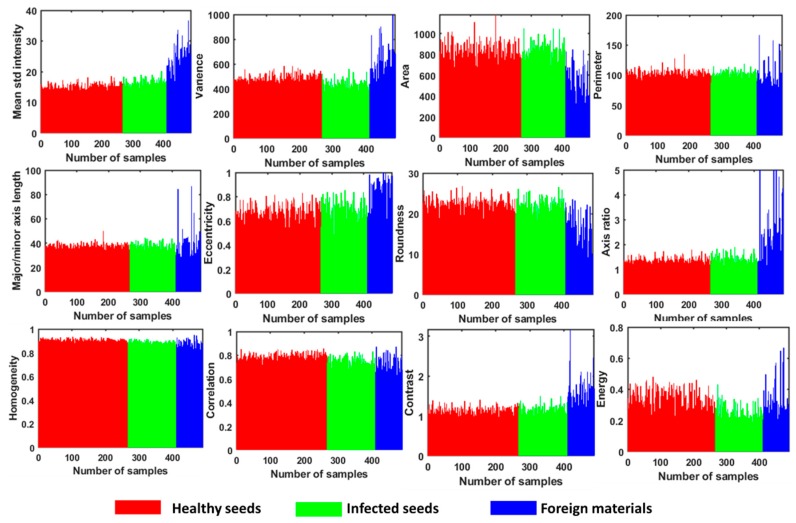
Color and textural feature extraction for tomato seed, foreign materials, and abnormal seed sample.

Feature selection for the identification of good tomato seeds of any variety from infected seed or foreign materials based on color, morphological, and textural features were used simultaneously. Hence, only color features for seed classification were not effective because some healthy seeds were light to dark brownish. Therefore, a combination of color features with morphological and textural characteristics is more reliable for classification parameters. These selected features were highly correlated with each other such that a single feature did not contribute significantly to the classification model. A sum of 14 features (2 color, 8 morphological and 4 textural features) is used to develop the classification model of healthy tomato seed identification and arranged in descending order of their level of contribution to the classification model, as shown in Figure 6. The features of 2000 healthy tomato seeds (1000 seeds for each variety) were initially extracted, and a calibration model was developed using multivariate data analysis combined with DD-SIMCA. The calibration model was developed using 2000 healthy tomato seeds with 14 features (2000 × 14 matrix) and was considered to be a target class.

Fourteen (14) accumulated features of the two varieties of healthy tomato seeds were evaluated. In the test, a total of 540 healthy seeds (270 seeds from each variety) were used to perform the one-way analysis of variance (ANOVA) test. As shown from the test results in Table 1, a significant difference (p = 0.0001) existed between the two varieties of healthy tomato seeds. Though there is significant difference between these two healthy seed varieties, seeds from any variety will be placed in one group for further classification purposes.

### 3.2. Seed Classification

To recognize healthy tomato seeds of any variety, all 14 (color, morphological and textural) obtained features for each of the 2000 healthy tomato seeds were accumulated to develop the calibration model by using multivariate data analysis combined with DD-SIMCA. Multivariate analyses can be used to develop the model by considering several features simultaneously. The OCC is an appropriate chemometric tool where the calibration step is only based on the target class, i.e., healthy tomato seeds from any variety in the study. Therefore, any sample out of boundaries was considered to be “external samples” (foreign material and infected seed). To establish the OCC, the pertinent number of principal components (PCs) were initially selected to minimize the prediction error that describes the most common characteristics of the investigated or target class. After establishing the number of PCs, the acceptance area was developed by estimating the DoF for score distance (SD) and OD. Hence, the method developed an agreement condition on the SD scales corresponding to the OD for a given value of type I error, i.e., α. The training set verified model performance on good tomato seeds. Seeds similar to the target or training class (healthy tomato seeds) were placed inside the acceptance area, and dissimilar seeds were placed outside the desired acceptance area. To evaluate model performance, the test set samples (including normal, abnormal, and broken tomato seeds with different featured external materials) that were closest to the cut-off area (acceptance plot) were used to calculate type II error, β.

**Figure 7:**
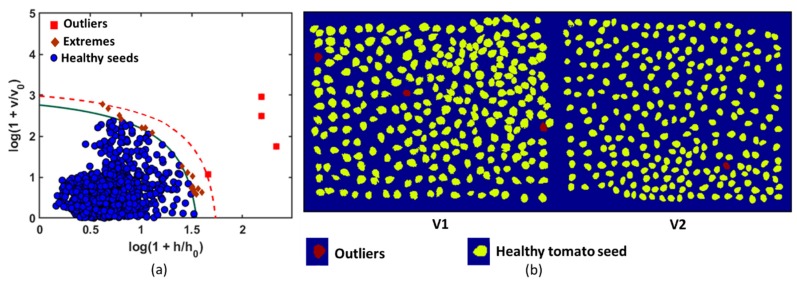
(**a**) Application of data-driven soft independent modeling of class analogy (DD-SIMCA) model calibration to identify for the target group authentication. The solid green curve limits the acceptance area (α = 0.001) and the dashed red curve limits the outlier area (γ = 0.05); (**b**) Healthy tomato seed identification using the calibration model for two varieties.

The optimal result was obtained with the first nine principal components (9 PCs). The agreement conditions were constructed using a robust chi-square distribution with SD = 7 and OD = 2 DoFs, which corresponded to the acceptance area of the calibration model as shown in Figure 5. The acceptance level of the model was considered to be very narrow (α = 0.001). The green solid curve was obtained for α and recognized the members of the training group (healthy tomato seeds). The region above the acceptance area (red broken line) represents the outlier area (outlier significance, γ = 0.05). In the present study, the DD-SIMCA model comprised a data training set with chi-square type acceptance area, a robust estimation procedure, and 9 PCs set into the model. The seeds that were set inside the acceptance area were considered to be target samples. The objects located outside the outlier area (broken red line) were classified as aliens. The model misclassified four seeds among 2000 healthy tomato seeds. Each sample included a separate individual number that was comprised of its features, and thus it was easy to identify the numbered sample that corresponded to an outlier. The samples with real seed samples were identified, and the samples were observed as darker in color and exhibited a different shape from that of the other healthy tomato seeds. Outliers from the calibration model were removed to decrease the misclassified number of healthy seeds in the test set. Hence, the ultimate goal of the study was to develop a calibration model and to accurately identify healthy seeds from a variety of intragroup groups (healthy seeds from infected seeds mixed with inert materials). Therefore, to optimize the dataset, the misclassified samples were removed, and the calibration model was recalculated. The color-coded image is generated to visualize healthy tomato seeds in a given color (yellow), and the outliers are denoted by another color (red), as shown in Figure 7.

**Figure 8:**
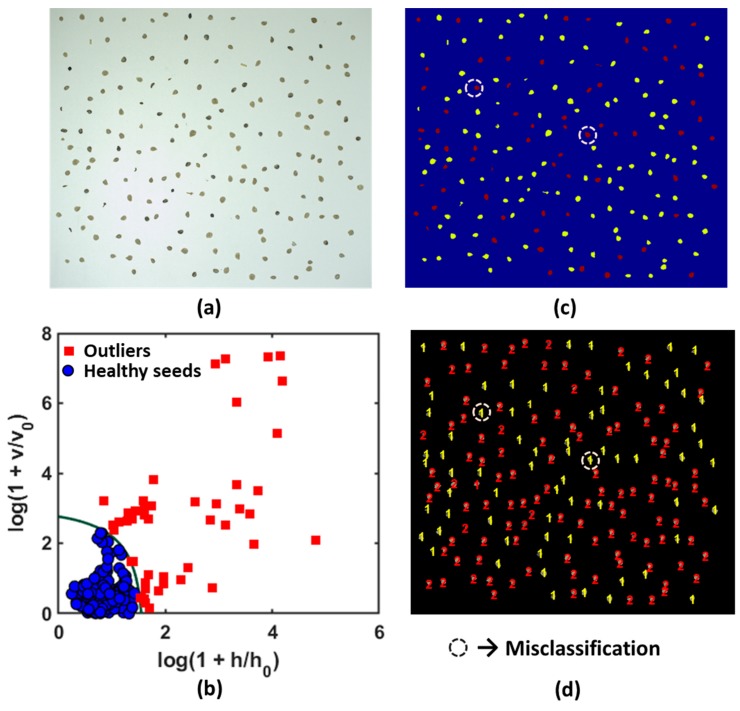
Test set classification with the developed model. (**a**) Original mixed samples; (**b**) Model application on test set; (**c**) Color coded samples; (**d**) Two groups were identified.

When the calibration model was developed, the validation stage was performed using a complex test set. In the test set, objects of the target class (i.e., seeds that were not used for model development; 1000 healthy seeds, 500 from each variety) with other mixed samples (different foreign materials) were included. The test set aided in evaluating model behavior to identify alien objects from the target class. The test sample set includes healthy tomato seed varieties and is mixed with infected/broken seeds and foreign materials as shown in Figure 8. The data for the test set were collected at different positions and orientations to evaluate the detection accuracy of the model. Both the samples and model were in good agreement, and the results indicated that the DD-SIMCA approach is highly sensitive in identifying foreign materials and infected/broken seeds from healthy tomato seeds. To validate the model, 1000 good seeds were grouped as 119 healthy seeds (V1→54, V2→65) and 63 inert materials (infected seeds→34, foreign materials→29) to cover the FOV. A total of 9 images were captured with different samples in different positions and orientations for the test sets. In Figure 8d, two groups of the test sample were identified visually, using the custom-built algorithm. 1→denotes the inert materials (including infected seed and foreign materials) and 2→denotes the healthy tomato seed from any variety. Two (2) healthy tomato seeds were misclassified as inert material in the validated model. All the 9 test images were analyzed in the similar way and recorded the data of misclassification of any group. After analyzing all the images using the developed calibration model, several healthy seeds were misclassified as infected seeds due to their shape and dark color. However, no infected seeds or inert material were misclassified as healthy tomato seeds. The goal of the study was to identify healthy seeds from inert materials accurately, and thus some healthy seeds can be discarded to fulfill the objective. All the test results were further analyzed to calculate other statistical parameters of the developed model to evaluate model performance.

An organic growing media germination test was performed to evaluate the germination capability for the seeds. After the germination test, it was found that less than (<) 10% of black-dotted seeds were shown germination capability whereas, the healthy seeds had provided greater than (>) 98% of germination rate. Therefore, discarding infected seeds from a healthy/good seed batch is a crucial criterion to increase crop yield.

**Figure 9:**
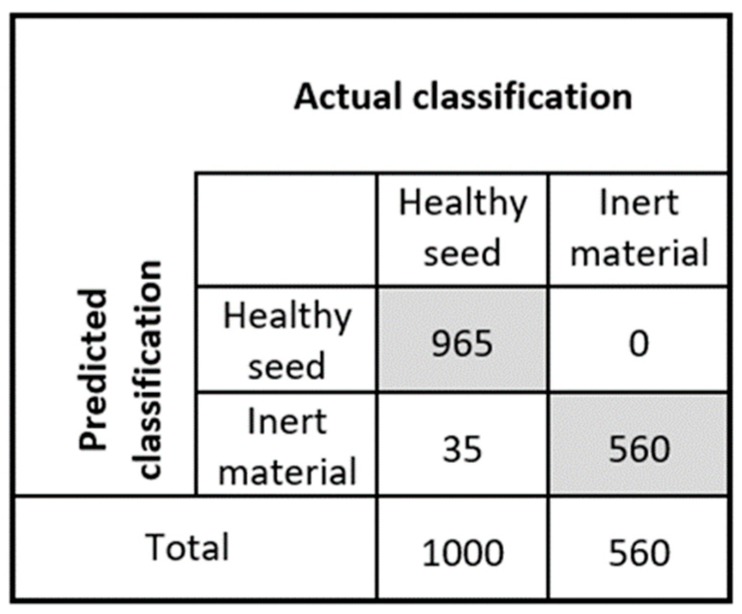
Confusion matrix of test data set from the developed model.

It is noted that the combination of image processing and DD-SIMCA multivariate data analysis leads to excellent results for the detection of foreign materials using a simple imaging technique. The discrimination of healthy tomato seeds from inert materials was performed successfully. However, some healthy seeds presented similar features to those of infected seeds. Hence, they were misclassified. The test sample detection result is demonstrated by the confusion matrix plotted in Figure 9, and the classification parameters are calculated.

As shown in Table 2, the results of the DD-SIMCA model for the validation set exhibit high sensitivity and specificity of 0.96 and 1, respectively, with an overall classification accuracy of 0.977. This implies that 96.5% of healthy seeds (965 out of 1000) were correctly classified and 100% samples of inert materials (560 out of 560) were accurately classified after applying the developed classification model. The model accuracy is 97.7%. Thus, the model can be accepted based on its final aim. The normal seeds that were misclassified as abnormal were darker in color when compared to the rest of the normal seeds. Hence, they were potentially considered to be abnormal seeds by the model.

Given the high accuracy of the proposed technique, the development of a similar interface for real-time healthy seed analysis is in progress. Furthermore, a sorting unit is synchronized with the developed machine vision-based sensing unit to physically separate healthy seed samples from inert materials. Hence, it can be considered to be the most practical detection method that offers fast, real-time, and large-scale inspection of individual seeds.

## 4. Conclusions

The study presented a machine vision-based efficient method for discriminating healthy tomato seeds from infected seeds and foreign materials. An image processing strategy incorporated with a multivariate data analysis method of DD-SIMCA was used to discriminate healthy tomato seeds from inert materials. Though the potential of proposed technique tested with only two varieties of tomato seeds in this work, its application can be extended to a wide range of varieties as well as to other crops’ seeds by developing an appropriate calibration model. The experimental results of the study indicated that DD-SIMCA in combination with the simultaneous use of various features can classify healthy tomato seeds from infected seeds or foreign materials with an accuracy of 97.7%. Additionally, a data acquisition algorithm was developed from a practical viewpoint. The proposed image processing methodology can be implemented in a software interface for rapid and real-time detection of healthy tomato seeds. This work paves the way for the development of a rapid, large-scale, and economically reasonable seed sorting system based on a machine vision technique, which will obviously exhibit high potential for the quality inspection of tomato seeds.

## Figures and Tables

**Table 1:** Statistical parameter difference between healthy seeds of two different varieties.

Sample	Sum of Squares	Mean Square	*F* Value	Prob > *F*
Healthy seeds of two varieties	1.31727 × 10^6^	1,317,268.8	14.92	0.0001*

* significantly different.

**Table 2:** Classification parameters from the test data set.

Test Set	Samples Used	Accuracy	Error Rate	Sensitivity	Specificity
Mixed	1560	0.977	0.023	0.96	1
